# Mid-thoracic Nodular Fasciitis: A Diagnostic Dilemma

**DOI:** 10.7759/cureus.64170

**Published:** 2024-07-09

**Authors:** Abeer Farhan, Abdulla Fakhro

**Affiliations:** 1 General Surgery, King Hamad University Hospital, Busaiteen, BHR; 2 Plastic and Reconstructive Surgery, King Hamad University Hospital, Busaiteen, BHR

**Keywords:** misdiagnosis, myofibroblastic proliferation, diagnostic challenge, soft tissue mass, nodular fasciitis

## Abstract

Nodular fasciitis (NF) is a rare, benign, yet rapidly proliferative myofibroblastic soft tissue tumor that often mimics malignant lesions and presents significant diagnostic challenges. This case report describes a 16-year-old female whose mid-thoracic mass was initially mismanaged in an emergency setting as a sebaceous cyst. The misdiagnosis and subsequent inappropriate incision and drainage led to an iatrogenic flare-up, exacerbating the patient's condition and complicating her management course. The complexities encountered in this case underscore the critical need for stringent diagnostic protocols and multidisciplinary management to avoid iatrogenic complications and improve clinical outcomes in patients presenting with unusual soft tissue lesions. This report highlights the importance of adhering to established protocols for soft tissue lump evaluation and the potential pitfalls of misdiagnosis.

## Introduction

Nodular fasciitis (NF) is a benign, self-limiting proliferative disorder involving the subcutaneous or superficial fascial layers, first described by Konwaller in 1955 as pseudosarcomatous fibromatosis [1]. NF typically presents as a rapidly growing, painless mass most commonly affecting young adults. It may occur anywhere on the body, but most commonly affects the forearm (27-29%) [2]. While the etiology of NF remains partially understood, it is believed to involve reactive proliferation possibly linked to minor trauma or inflammation [3]. This rapid and sometimes alarming growth often leads to misdiagnoses as more aggressive or malignant lesions, emphasizing the need for accurate diagnosis to prevent unnecessary patient anxiety and inappropriate interventions.

## Case presentation

**Figure 1:**
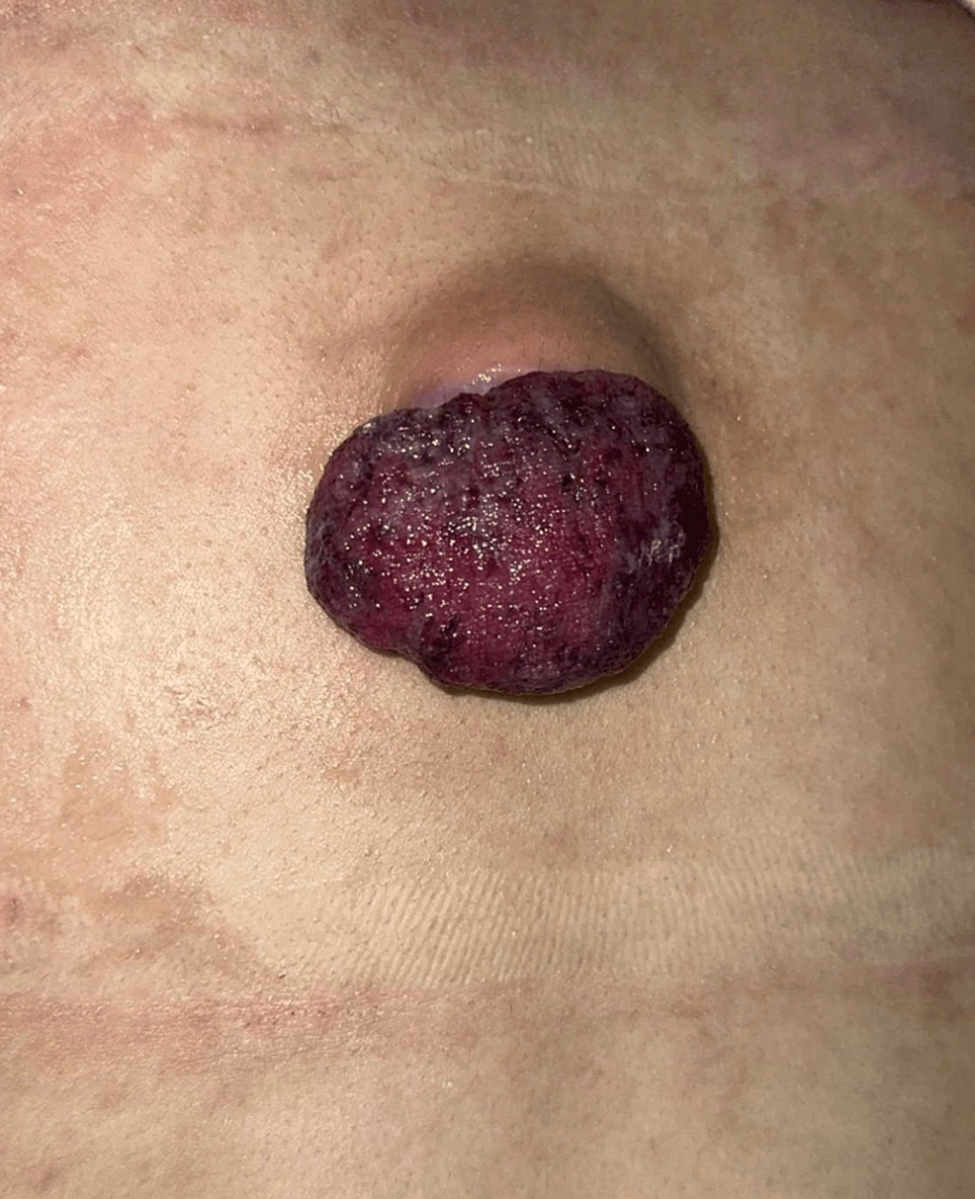
Pre-operative image displaying protruding erythematous cauliflower-like mass over mid-thoracic region at I&D site I&D: Incision and drainage

A 16-year-old female presented with a month-long history of a slowly enlarging mass on her mid-back. Initial treatment by emergency department personnel involved incision and drainage under the assumption of an infected sebaceous cyst, which yielded a gelatinous material. Post-procedure, the lesion transformed into a rapidly enlarging, cauliflower-like growth at the surgical site (Figure 1).

**Figure 2:**
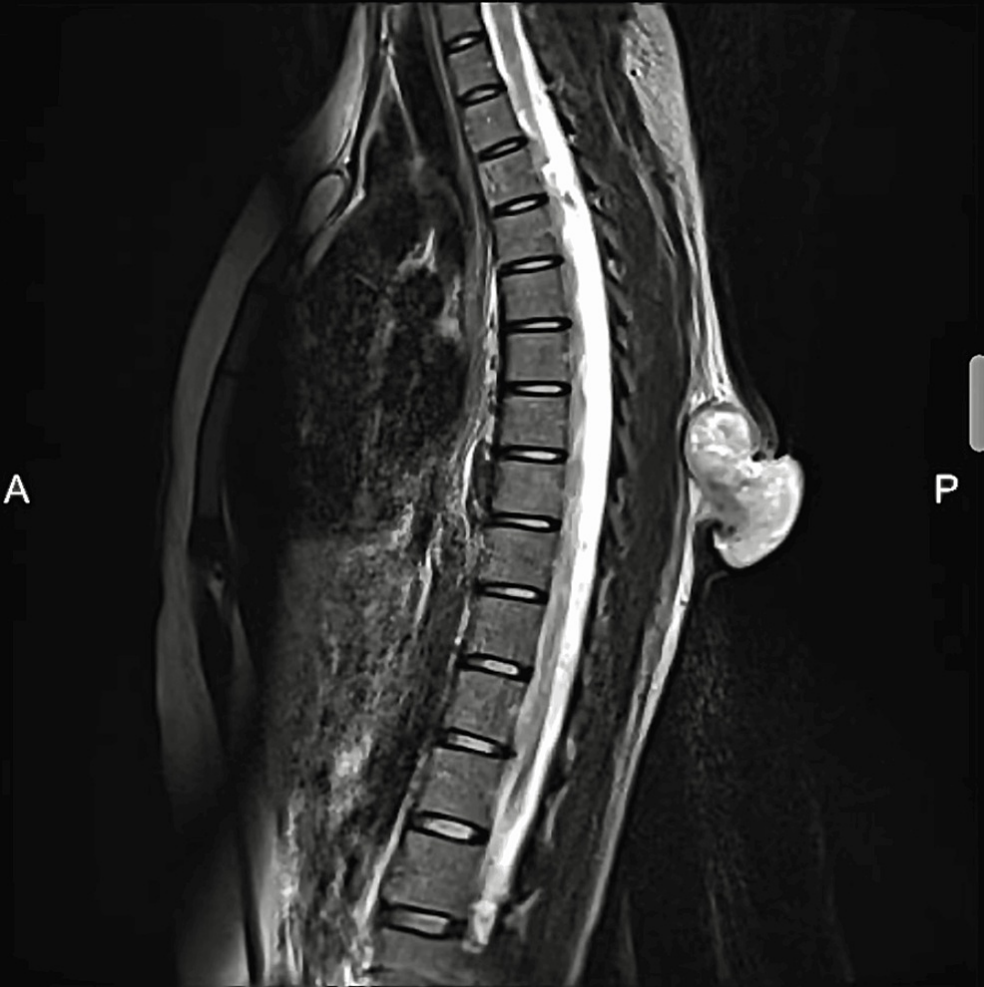
MRI spine sagittal T2 view showing a heterogenous exophytic mass over the subcutaneous soft tissue plans at posterior midline mid thoracic with contrast enhancement suspicious for infiltration

Histopathological examination from an excision biopsy initially suggested a low-grade fibromyxoid sarcoma. Subsequently, an MRI of the spine exhibited an exophytic soft tissue lesion with lobulated contours and a hypervascular component measuring 38 x 55 x 55 mm located in subcutaneous soft tissue plans in the posterior mid-thoracic region (Figure 2).

The patient was scheduled for an excision biopsy by general surgery. She was referred to our plastic and reconstructive clinic for possible reconstruction of the residual defect. The patient was informed about her reconstructive alternatives, including a lower trapezius fasciocutaneous flap versus a scapular flap. Following excision, primary closure of the 5 x 7 cm defect was successfully achieved without necessitating additional tissue manipulation or local reconstruction. This facilitation of closure can be attributed to the intrinsic tissue expansion effect exhibited by these soft tissue tumors, which enhances the mobilization of the surrounding soft tissue envelope. The phenomenon is analogous to the deliberate expansion induced by a surgical tissue expander, effectively increasing the available tissue for closure. The patient was discharged the next morning.

**Figure 3:**
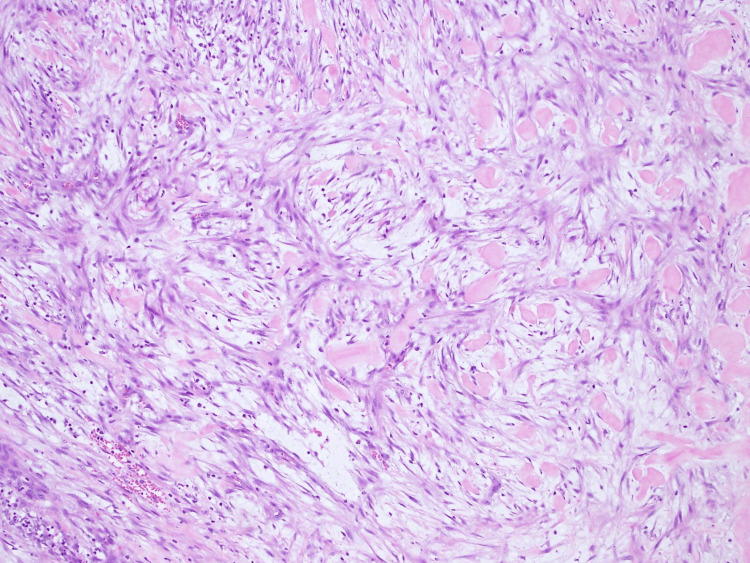
Microscopic examination with H&E Stain depicts haphazard proliferation of bland myofibroblastic spindled cells in tissue culture arrangement with multifocal microcystic changes in myxoid stromal background with scattered erythrocytes

The final pathology report exposed haphazard proliferation of bland myofibroblastic spindled cells with multifocal microcystic change and an associated inflammatory cell infiltrate (Figure 3). Positive immune reactivity for SMA and CD10, while negative for desmin, S100, and CD34, verified NF. The patient was followed up with no evidence of recurrence.

## Discussion

NF can typically present as a solitary, painless proliferative nodule affecting individual adults between the ages of 20 and 40 [4,5]. The exact biological behavior remains an area of investigation. However, the proliferative nature has been elucidated by the identification of recurrent gene rearrangements of ubiquitin-specific protease 6 (USP6) in approximately 90% of NF cases [6].

On imaging, particularly MRI, NF typically appears with a homogeneous low signal intensity on T1-weighted images and a heterogeneous intermediate signal intensity on T2-weighted images, accompanied by surrounding edema and slightly uneven enhancement [7]. It may also display aggressive characteristics such as crossing compartment boundaries and involving bones or joints. The imaging characteristics are often non-specific, making it challenging to differentiate NF from sarcoma solely based on imaging results. Consequently, NF is usually distinguished from sarcoma through immunohistochemical analysis.

**Figure 4:**
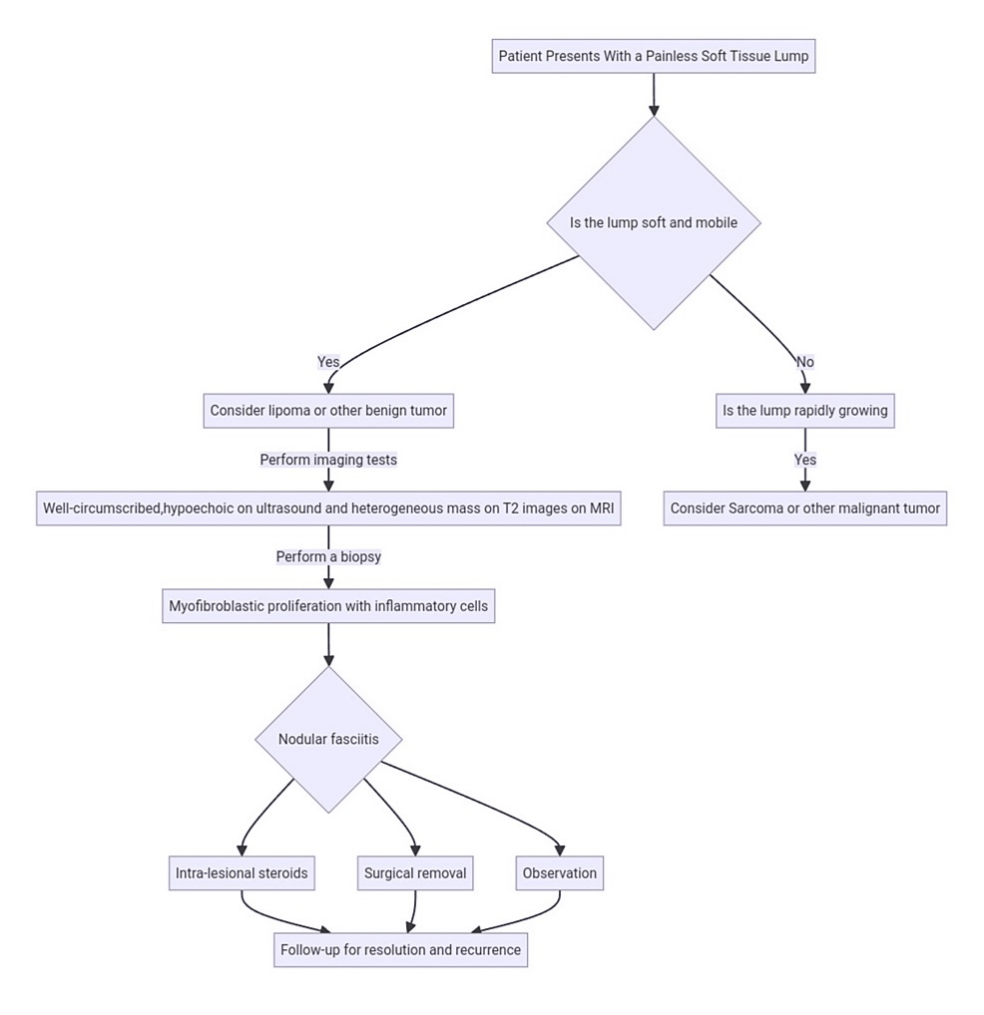
Diagnostic algorithm for the evaluation of painless soft tissue mass

In light of the fundamental principles of soft tissue lump management (Figure 4), it appears that the care pathway for this patient could have been more carefully considered. Preoperative imaging, typically pivotal in such cases, was not utilized, and the decision to proceed with drainage was made despite the absence of definitive signs of abscess formation. Furthermore, the presence of gelatinous myxomatous material during the incision, a critical diagnostic observation, was not adequately addressed. This oversight may have inadvertently contributed to a series of inflammatory reactions, culminating in the release of growth factors and the development of the cauliflower mass.

A complete, wide surgical excision represents the gold standard for both diagnostic and curative purposes [8]. Spontaneous regression and rapid resolution with intralesional corticosteroid injection have been reported [8]. Radiotherapy is considered a last resort for lesions involving vital organs, such as the eyes [9]. Recurrence is generally rare; however, in cases of recurrence, some studies have reported the effective use of targeted steroid injections [8].

## Conclusions

This case of NF, exacerbated by iatrogenic injury, underscores the importance of comprehensive preoperative evaluation, including appropriate imaging like ultrasonography, to guide clinical management. It highlights the need for healthcare providers to be familiar with NF and adhere to established diagnostic protocols to ensure optimal patient care. Additionally, it demonstrates the value of multidisciplinary collaboration, particularly involving plastic and reconstructive surgeons, in managing complex soft tissue lesions to prevent misdiagnosis and ensure effective treatment outcomes.
